# Blastocysts Derived From 0PN Oocytes: Genetic And Clinical Results

**DOI:** 10.5935/1518-0557.20190084

**Published:** 2020

**Authors:** Maria Valeria Paz, Mariel Chiera, Paula Hovanyecz, Juliana Cicaré, Patricia Perfumo, Luciana Domenech, Viviana Ventura

**Affiliations:** 1 Servicio de Medicina Reproductiva, Instituto Gamma, Rosario, Argentina

**Keywords:** 0PN, implantation rate, fertilization failure, blastocyst, diploidy

## Abstract

**Objective:**

To analyze the genetic and clinical outcomes of blastocysts derived from 0PN oocytes after IVF/ICSI.

**Methods:**

This retrospective observational study included patients aged 40 years or younger submitted to IVF/ICSI with their own oocytes and with blastocysts derived from 0PN oocytes between January 2015 and April 2018. The clinical outcomes of 0PN blastocyst transfers were analyzed. Genetic tests were performed on biopsied 0PN blastocysts with Next Generation Sequencing.

**Results:**

A total of 27 0PN blastocysts were transferred, yielding an implantation rate of 48.0% and an ongoing pregnancy rate of 50.0%. The transfers resulted in 13 live births (59.0% live birth rate). Genetic test results revealed that four of the 17 0PN blastocysts biopsied were 46XX; three were 46XY; and 10 were aneuploid embryos, awarding a diploid rate to 76.4% (13/17).

**Conclusion:**

Almost half of the 0PN blastocysts implanted (48.0%) and 13 healthy babies were born. More than three quarters (76.4%) of the 0PN blastocysts were diploid, thus ruling out the possibility of parthenogenetic activation. Our study indicated that the transfer of 0PN blastocysts is a safe, worthy option when the number of normal 2PN embryos is insufficient.

## INTRODUCTION

The assessment of fertilization on in vitro fertilization (IVF) or intracytoplasmic sperm injection (ICSI) procedures is generally performed at a precise moment after insemination/injection (18±2 hours). An oocyte is deemed fertilized when two pronuclei (2PN) and two polar bodies are viewed after insemination. It is known that 20-30% of mature oocytes do not show evidence of fertilization (no pronuclei visible, 0PN) under light microscopy (Feenan & Herbert, 2006). Although some 0PN cleaved oocytes may be seen on day 2, the ESHRE Guidelines do not recommend transferring these embryos (ESHRE Guideline Group on Good Practice in IVF, 2016). Since at times an insufficient number of good quality 2PN embryos is available, one might wonder whether 0PN embryos with normal morphology may be transferred. Burney *et al*. (2008) and Li *et al.* (2015) reported births from 0PN embryo transfers. Li *et al.* (2015) also compared the outcomes of 0PN and 2PN embryo transfers at the cleavage stage *vs.* the blastocyst stage. The authors found that 0PN implantation rates were lower than 2PN cleavage stage transfers, but not compared with blastocyst stage transfers (Li *et al*., 2015). However, few papers have reported on 0PN embryo transfers resulting in healthy babies (Burney *et al*., 2008; Manor *et al*., 1996; Li *et al*., 2015; Destouni *et al*., 2018).

On the other hand, there is much controversy concerning the genetic constitution of 0PN embryos. Authors analyzing the ploidy status of 0PN embryos reported cases of diploidy, thus ruling out the possibility of parthenogenetic activation (Manor *et al*., 1996; Lixin *et al*., 2014).

In our center, we have observed that a significant number of 0PN oocytes cleave (27%) and 35% reach the blastocyst stage. Therefore, our study aimed to retrospectively analyze the clinical outcomes of transfers of blastocysts derived from 0PN oocytes after IVF/ICSI performed in our center, and examine the genetic constitution of 0PN blastocysts.

## MATERIAL AND METHODS

### Patients

Our study included patients aged 40 years or younger submitted to IVF/ICSI procedures using their own oocytes and with blastocysts derived from oocytes without evidence of fertilization (0PN). The study was carried out between January 2015 and April 2018.

Controlled ovarian stimulation and oocyte retrieval were performed according to previously published protocols, in accordance with patient clinical history and response to therapy (La Marca & Sunkara, 2014).

### Laboratory protocols

Oocytes, semen preparation, and IVF/ICSI procedures have been extensively described (Sepúlveda et al., 2009; Ebner et al., 2001). The embryos were individually cultured in 25µ drops under mineral oil. Incubators with low oxygen tension were used (6% CO_2_, 5% O_2_ and 89% N_2_) (Paz et al., 2017). Fertilization was deemed normal when two pronuclei were seen 16-20 h after insemination. However, oocytes without signs of fertilization (0PN) were kept under observation. Embryonic divisions were observed on day 2 (41-44 h post insemination) and were graded from 1 to 4 according to the Istanbul consensus (Paz *et al*., 2017; Alpha Scientists in Reproductive Medicine & ESHRE Special Interest Group of Embryology, 2011). 0PN oocytes undergoing cell division were kept on culture until they reached the blastocyst stage. On day 5, the blastocysts were graded based on the system developed by Gardner and Schoolcraft, taking into account the morphology of the internal cell mass (ICM), the trophectoderm (TE), and the degree of blastocoel expansion (Alpha Scientists in Reproductive Medicine & ESHRE Special Interest Group of Embryology, 2011; Gardner & Schoolcraft, 1999).

### Embryo transfer

Fresh or cryopreserved blastocyst transfers were performed on days 5/6 under ultrasound guidance, after the best embryos were selected based on morphology assessment. Good quality blastocysts derived from 2PN oocytes were selected for transfer, but in cases where no other blastocysts were available, the 0PN were chosen for transfer.

Mixed double transfers of 0PN and 2PN blastocysts forming only one gestational sac were excluded, since it is not possible to determine which of the two implanted.

### Blastocyst biopsy

The embryos underwent laser-assisted hatching (Lykos, Hamilton Thorne Inc., USA) on day 3 of development. Only blastocysts with good-quality ICM and TE were biopsied on day 5 or 6 of development based on grading. Each blastocyst was placed in a 25-µl drop of buffered medium and covered with paraffin oil. The hatching of the zona pellucida and the TE was performed at the 3 o’clock position, and gentle suction was applied to the blastocyst via a holding pipette (Origio, USA). A biopsy pipette (Origio, USA) was used to gently aspirate the trophectoderm into the bore of the needle. Laser pulses (400 µs) were used to remove the TE.

### Outcome parameters

The following clinical outcomes were analyzed in our study: embryo transfer clinical pregnancy rate (CPR); implantation rate (IR); embryo transfer ongoing pregnancy rate (OPR); and embryo transfer live birth rate (LBR) (Zegers-Hochschild *et al.,* 2017).

Clinical pregnancy was confirmed by visualization of a gestational sac five weeks after embryo transfer. IR was defined as the number of gestational sacs per 0PN embryo transferred. The status of ongoing pregnancy was assigned for pregnancies lasting ≥ 20 weeks. LBR was defined as the number of deliveries that resulted in live births expressed as a percentage. All infants were evaluated for signs of complication.

In order to evaluate the diploid state of 0PN embryos and rule parthenogenetic activation (haploidy) out, the results of next generation sequencing (NGS) of the 0PN blastocysts from the patients admitted to the PGT-A program were analyzed (Ilumina Platform, Coopergenomics) (Scott *et al*., 2013).

## RESULTS

A blastulation rate of 51% was observed in the study (1701 blastocysts from 3310 embryos). Seventy-eight blastocysts obtained from 58 patients submitted to IVF/ICSI were derived from 0PN oocytes, the equivalent to 2% of the total blastulation rate (78 0PN blastocysts from 3310 embryos).

A total of 22 0PN blastocyst transfers were made (five fresh and 17 frozen-thawed embryos). Nine single-embryo transfers (1 0PN blastocyst), five pure double embryo transfers (2 0PN blastocysts), and eight mixed double transfers (0PN/2PN blastocysts with 2 gestational sacs) were performed, resulting in respective ongoing pregnancy rates of 33.3%, 60.0% and 62.5% (Table 1). The overall OPR was 50.0%. A total of 27 0PN blastocysts were transferred, yielding an IR of 48.0% (13 gestational sacs from 27 0PN blastocysts). All ongoing pregnancies came to term, resulting in 13 healthy live births (59.0% LBR). The remaining 51 blastocysts are still cryopreserved.

**Table 1:** Clinical Outcomes of 0PN Blastocyst Transfers

	Nº ET	Nº 0PN Embryos Transferred	CPR	IR	OPR	Deliveries	LB 0PN
**Simple ET (1 0PN)**	9	9	33.3% (3/9)	33.3% (3/9)	33.3% (3/9)	3	3
**Pure double ET (2 0PN)**	5	10	60.0% (3/5)	50.0% (5/10)	60.0% (3/5)	3	5
**Mixed double ET (0PN/2PN)**	8	8	62.5% (5/8)	62.5% (5/8)	62.5% (5/8)	5	5
**Total**	**22**	**27**	**50.0% (11/22)**	**48.0% (13/27)**	**50.0% (11/22)**	**11**	**13**

ET: embryo transfer;

CPR: clinical pregnancy rate;

IR: implantation rate;

OPR: ongoing pregnancy rate;

LB: live birth

Twelve patients in the group of 22 transfers performed in our study had been previously submitted to embryo transfers with 2PN embryos, but were unable to achieve ongoing pregnancy.

Seventeen 0PN blastocysts from 13 patients were biopsied in preimplantation genetic testing for aneuploidies. The reasons for undergoing PGT-A were advanced maternal age (7), recurrent miscarriage (2), repeated implantation failure (2), and patient choice (2). NGS outcomes were: four euploid 46XX embryos; three euploid 46XY embryos; and 10 aneuploid embryos with different monosomies or trisomies. More than three quarters (76.4%; 13/17) of the 0PN embryos were diploid (three 46XY and 10 aneuploid embryos) and unrelated to parthenogenetic activation (since the 2PNs were not displayed). A diploid state could not be confirmed for the four 46XX embryos, since the employed technique has its limitations at detecting complete balanced haploidies (it cannot distinguish 23X from 46XX).

## DISCUSSION

Transfers of blastocysts derived from 0PN oocytes yielded an IR of 48.0% and an OPR of 50.0%. Double transfers - pure double ET (2 0PN) and mixed double ET (0PN/2PN) - produced similar outcomes. In our study, the transfers of 0PN blastocysts led to the birth of 13 healthy babies.

The presence of oocytes without pronuclei (0PN) is not always an indicator of fertilization failure. A proportion of 0PN is able to cleave and form embryos with similar morphology to 2PN embryos. The cleavage of 0PN zygotes may result in normal or abnormal fertilization (1PN or > 2PN) or parthenogenetic activation (Feenan & Herbert, 2006). Manor *et al*. (1996) analyzed some chromosomes using fluorescence in situ hybridization in 23 0PN embryos and found that 57% of the 0PN embryos contained a normal diploid chromosomal complement, indicating that the origin of these embryos was not parthenogenetic activation, but fertilization in which pronuclei were not observed. Destouni *et al.* (2018) used genome-wide haplotyping to study 0PN and 1PN diploid embryos to find whether they had uniparental diploidies. The authors found bi-parental diploidy in 75.5% of the 0PN and 42.86% of the 1PN embryos. Since reported euploidy rates were similar among 0PN, 1PN and 2PN embryos, their use in IVF is being discussed (Destouni *et al*., 2018). Our genetic tests found that 76.4% of the 0PN blastocysts were diploid, but we were unable to rule out possible uniparental diploidies.

Pronuclear membrane breakdown may occur at different times. Time Lapse (TL) technology allows the accurate observation of pronuclei at any time. Different studies using TL showed that pronuclei might appear at different times, from as early as 6.16 h to as late as 29.4h after microinjection (Basile & Meseguer, 2012; Basile *et al*., 2013). This indicates that oocytes without pronuclei at the time of fertilization check (16-20 h after insemination) may actually be zygotes with 2PN in which the pronuclei either disappeared before fertilization check or will appear after some time.

It is difficult to determine whether a 0PN embryo has originated from normal or abnormal fertilization (1PN, >2PN) or parthenogenetic activation. 0PN embryos originated from normal fertilization with retarded or advanced formation of pronuclei may yield near-normal implantation and pregnancy rates. Cleavage embryos originated from abnormal fertilization (Wang *et al*., 2012) and parthenogenetic activation (Yan *et al*., 2011) yield low blastulation rates. Li *et al.* (2015) reported lower implantation rates from 0PN cleavage stage embryos compared to 2PN embryos, but when blastocyst stage embryos were transferred, similar rates were obtained for 0PN and 2PN blastocysts.

Although TL may overcome the limitations of conventional fertilization checks, Capalbo *et al*. (2017) showed that even when the PN state is assigned by TL, four in five 1PNs are diploid. This indicates that pronuclear evolution might not always reflect bi-parental diploidy, and that even when the PN status (together with PB scoring) is verified with TL, a subset of embryos are subject to misclassification.

The protocol in effect in our laboratory dictates that embryos derived from 0PN oocytes undergoing cell division be cultured to the blastocyst stage, a procedure that facilitates the selection of normal 0PN embryos for transfer or cryopreservation. The 13 healthy babies born in our study have increased the yet small set of studies reporting healthy live births from 0PN embryos (Liu *et al*., 2016; Burney *et al*., 2008; Manor *et al*., 1996; Li *et al*., 2015; Destouni *et al*., 2018). In this sense, our data strengthens the recommendation not to discard 0PN-derived embryos.

## CONCLUSION

Many assisted reproduction centers do not transfer unfertilized oocytes. However, for a specific group of patients - particularly individuals with poor ovarian response or repeated implantation failure - it might be advisable to culture 0PN oocytes to the blastocyst stage and consider them as safe enough for transfer, to thus offer these patients a chance of having additional transfers and possibly achieving pregnancy.
